# Chronic Wasting Disease—Prion Disease in the Wild

**DOI:** 10.1371/journal.pbio.0020121

**Published:** 2004-04-13

**Authors:** Steve Bunk

## Abstract

Chronic wasting disease in deer is the only prion disease that infects both free-ranging and captive animals -- a situation that greatly complicates efforts to control it

In 1967, mule deer in a research facility near Fort Collins, Colorado, in the United States apparently began to react badly to their captivity. At least, that was the guess of researchers working on the natural history and nutrition of the deer, which became listless and showed signs of depressed mood, hanging their heads and lowering their ears. They lost appetite and weight. Then they died—of emaciation, pneumonia, and other complications—or were euthanized. The scientists dubbed it chronic wasting disease (CWD), and for years they thought it might be caused by stress, nutritional deficiencies, or poisoning. A decade later, CWD was identified as one of the neurodegenerative diseases called spongiform encephalopathies, the most notorious example of which is bovine spongiform encephalopathy (BSE), more commonly known as mad cow disease. Nowadays, CWD is epidemic in the United States. Although no proof has yet emerged that it's transmissible to humans, scientific authorities haven't ruled out the possibility of a public health threat. The media have concentrated on this concern, and politicians have responded with escalated funding over the past two years for fundamental research into the many questions surrounding this mysterious disease.

Quite apart from how little is yet known about CWD, media interest is reason enough to step up investigation of it, says Mo Salman, a veterinary epidemiologist at Colorado State University in Fort Collins. He's been scientifically involved with BSE, since it was first discovered among cattle in the United Kingdom in 1986. He recalls predicting that lay interest in BSE would wane after five years. Instead, the disease was found in the mid-1990s to be capable of killing humans who ate tainted beef. “I was wrong, and it really changed my way of thinking, to differentiate between scientific evidence and the public perception,” Salman admits. “Because CWD is similar to BSE, the public perception is that we need to address this disease, to see if it has any link to human health.”

*CWD is the only spongiform encephalopathy known to naturally infect both free-ranging and captive animals, a situation that greatly complicates efforts to monitor, control, or eradicate it.*

## Increasing Attention

In 2001, the United States' Department of Agriculture (USDA) declared an emergency after CWD was first diagnosed in deer east of the Mississippi River, indicating a potential nationwide problem. This year, the USDA is developing a herd certification program to help prevent the movement of infected animals in the game farming industry. This will bolster monitoring already underway in virtually every state, including postmortem examinations of game killed by hunters and by sharpshooters in mass culling operations.

By June 2003, brain tissue from more than 111,000 animals had been sampled in North America, and 629 were found to have tested positive for CWD. That's a small epidemic compared to the thousands of BSE cases detected in cattle in the United Kingdom, but CWD is thought to be slow-spreading and perhaps lurking undiscovered elsewhere. So far, the United States and Canada are the only countries in which it has been identified, apart from a few imported cases in the Republic of Korea, but surveillance has not been thorough in North America and is virtually nonexistent in the rest of the world.

Considered 100% fatal once clinical signs develop, CWD has struck three species of the cervid family—mule deer, white-tailed deer, and Rocky Mountain elk—which roam wild and are raised on farms for meat and hunting. It's the only spongiform encephalopathy known to naturally infect both free-ranging and captive animals, a situation that greatly complicates efforts to monitor, control, or eradicate it.

The economic costs are hard to quantify, but a 2001 survey by the United States Department of Commerce's Bureau of Census shows that big-game hunters nationwide spend more than US$10 billion annually for trips and equipment. By far, their main target is deer. Wildlife watching of large land mammals, principally deer, drew 12.2 million participants in 2001. The North American Deer Farmers Association represents owners of 75,000 cervid livestock raised for their meat and for velvet antler, a health-food supplement made from antlers. These animals are valued at more than US$111 million.

**Figure 1:**
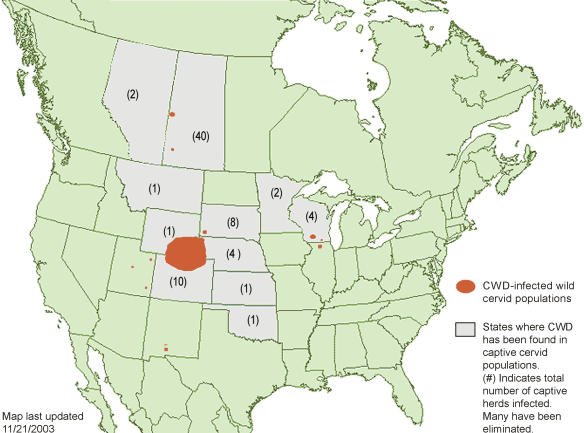
CWD in North America CWD has been detected in both free-ranging and captive animals in Wyoming, Colorado, South Dakota, Wisconsin, and Saskatchewan; only in captive herds in Montana, Nebraska, Kansas, Oklahoma, Minnesota, and Alberta; and only in wild animals in New Mexico, Utah, and Illinois. (Figure courtesy of Gary Wolfe and the CWD Alliance.)

Over the past two years, the federal government's emphasis on CWD has been “quite high” compared to other wildlife diseases, says USDA staff veterinarian Dan Goeldner. “In no small part, that's because the disease has cropped up in new places, and those are states that have political clout.” It has now been found in ten more states beyond what became known as the endemic region of Colorado and neighboring Wyoming (Figure 1). Last year, the USDA received US$14.8 million to monitor and manage the disease, and Goeldner says the department expects to get about US$16 million this year.

## The Prion Diseases

Those figures don't include scientific research funded by other organizations, such as the US$42.5 million received by the United States' Department of Defense in 2002 to start up a National Prion Research Program. The prion is the protein-like agent that causes transmissible spongiform encephalopathies (TSEs). Its normal function is uncertain, but when it misfolds into an abnormal or “infectious” form, it causes the microscopic holes and globs of toxic, misshapen protein found in the brains of TSE victims. Unlike viruses, prions don't contain nucleic acids—only protein. Without DNA or RNA to issue biochemical commands, abnormal prions shouldn't be able to convert normal prions to the infectious state, but that's exactly what they do (Box 1).

Prion diseases occur in many species. In domestic sheep and goats, prion diseases occur as scrapie, which has a virtually worldwide distribution. North America and Europe have also reported rare cases of TSE in ranched mink. Humans get kuru, Creutzfeldt-Jacob disease (CJD), and Gerstmann-Sträussler-Scheinker syndrome—all rare—and BSE itself manifests in people as a variant form of CJD. Since the United Kingdom outbreak, BSE has been discovered in more than 20 countries, most recently in North America. As public fear rose of possible CWD transmission to humans who eat infected venison, the United States' Centers for Disease Control and Prevention (CDC) released a report last year of its investigation into several deaths among venison eaters who might have had a TSE. The report concluded that none of the deaths could be attributed to venison, but it nevertheless cautioned that animals showing evidence of CWD should be excluded from the human and animal feed chains (Box 2).

CWD is the least understood of all the prion diseases. Its origins are unknown and may well never be discovered. The question is largely academic, unless one hypothesis is proven true, that it derives from scrapie. In that case, the knowledge might help in efforts to control the two diseases through herd and flock management.

Researchers are working to determine the minimum incubation time of CWD before clinical signs appear, now roughly estimated at 15 months in deer and 12—34 months in elk. They're trying to discover whether CWD strains exist that can affect the length of the disease process and different regions of the brain or that can infect different species, including humans. They are also investigating the period during which the prion is passed on, as well as its modes of transmission. They want to know whether disease reservoirs exist in the bodily fluids of hosts, in the environment, or both. They're racing to develop a diagnostic test that can be performed on live animals, enabling identification of the disease before clinical signs appear, which would eliminate the need to kill thousands of apparently healthy animals in areas where CWD is detected. But among the first things they need to clarify are CWD's distribution across North America and its prevalence.

*“…the disease has cropped up in new places, and those are states that have political clout.”*

## An Initial Step: Improved Surveillance

“Before you can start to control CWD, you need to understand where it is and how much of it you have,” says veterinary pathologist Beth Williams of the University of Wyoming in Laramie. “So I think you really need surveillance.” Research on its pathogenesis and transmission will help to develop better diagnostic tools, which will improve surveillance, adds Williams, who first identified the disease as a TSE more than a quarter-century ago.

Colorado State's Salman argues that current surveillance is primarily a series of reactions to reported cases, rather than a systematic strategy designed to determine where and at what prevalence the disease exists and where it's absent. The estimated prevalence is about 1% in elk and 2.5% in deer. But Salman says, “We don't have a good idea of areas in which we are saying we haven't found the disease because these areas are not yet, in my estimation, negative for the disease. Scrapie is a wonderful example of systematic surveillance but, to be fair to the decision-makers and technical people involved with CWD, surveillance on wildlife species is very difficult.”

The USDA's Goeldner declares, “We have the goal and the hope to eradicate the disease from the farm population.” But Colorado Department of Wildlife veterinarian and CWD expert Mike Miller warns, “Given existing tools, it seems unlikely that CWD can be eradicated from free-ranging populations once established.”

The gold standard of diagnosis is based on examination of the brain for spongiform lesions and abnormal prion aggregation. Suspect animals are decapitated and their bodies incinerated. “This is an approach that nobody wants, including the people who have to implement it,” says wildlife ecologist Michael Samuel, principal investigator in the United States Geological Survey–Wisconsin Cooperative Wildlife Research Unit at the University of Wisconsin in Madison.

Nevertheless, when three white-tailed deer shot by hunters in the south-central part of that state during the fall of 2001 were diagnosed with CWD, the state government took swift action. By the spring of 2003, almost 40,000 deer had been sacrificed and sampled for the disease, both within and without a 411 square-mile (1065 square-kilometer) region dubbed the eradication zone. There the goal was to remove as many deer as possible, whereas the plan in contiguous outlying areas was to reduce density to about ten deer per square mile. CWD is thought to spread more efficiently in high-density populations, and normal densities in Wisconsin are 50–100 deer per square mile, about five times that of Colorado and Wyoming. The main objectives of the Wisconsin culling were to discover where the disease existed and its prevalence in affected areas. In the eradication zone, it was 6%–7%, although in the outlying region it was only 1%–2%. Samples elsewhere in the state tested negative.

## In Search of a Live Assay

A key to combating the spread of CWD is to put into widespread use a preclinical diagnostic test on live animals. Miller and colleagues recently developed and validated the first such assay, based on a biopsy of lymphoid tissue, where the infectious agent is known to incubate. They showed that tonsillar biopsies taken from live animals can confirm disease at least 20 months prior to death and up to 14 months before the onset of clinical signs. Although the method is a useful screening tool, it requires much time and training. Each deer must be anaesthetized and blindfolded, placed in a restraint, its mouth held open with a gag, the tonsil visualized with a laryngoscope, and the biopsy taken with endoscopic forceps. Lymphoid tissue sampling was first used as a preclinical test in sheep scrapie. “Many attempts have been made to develop and evaluate tests for live animals, but it is fraught with difficulties,” declares TSE specialist Danny Matthews of the United Kingdom Government's Veterinary Laboratories Agency in Weybridge. He says that a live test for BSE in cattle is likely to be evaluated shortly by the European Food Safety Authority, but warns of a major problem: test samples are collected early in the incubation, whereas brain pathology only arises two to three years later. This creates long delays in determining whether a positive preclinical test result is, in fact, accurate: “How can one do an appropriate evaluation?”

Matthews notes that blood appears to be a useful medium for testing scrapie in sheep, but current technology cannot deliver a tool applicable across a range of different scrapie genotypes. “Like sheep, elk and mule deer do have a peripheral pathogenesis, which suggests that the blood test route may have some potential, especially if the genotype variability is more restricted than in sheep.”

## Transmission Mysteries

Scrapie can be vertically transmitted from mother to offspring, either in the womb or from the transfer of infected germ plasm. It also can be transmitted horizontally, from any one animal to another. CWD, the only other known contagious TSE, is thought to be transmitted solely by as-yet-undetermined direct or indirect horizontal contact. It probably is not transmitted through infected feed, as is the case for BSE.

A number of scientists are currently on the trail of suspected CWD disease reservoirs. Saliva is a leading candidate, because clinical signs of CWD include excessive thirst, drinking, and drooling. Work with lab animals suggests that the infectious agent might be produced in salivary glands and, if so, it could be transmitted through social interactions. Feces is also a possible reservoir because animals nose in the ground for feed, and urine is yet another candidate, because it is involved in the scenting activities of cervids.

Soil could be an environmental reservoir, because cervids ingest dirt to supplement their diets with minerals. Bucks also lick soil on which does have urinated to ascertain their mating status. University of Wisconsin soil science professor Joel Pedersen has discovered that abnormally folded prions stick to the surface of some soil types, such as clay, resisting environmental and chemical damage. “Captive elk contracted CWD when introduced into paddocks occupied by infected elk more than 12 months earlier, despite fairly extensive efforts to disinfect the enclosures,” Pedersen notes. He has begun a five-year project to characterize interactions between infectious prions and soil particles and determine the extent to which infectivity is retained.

No matter how CWD is transmitted between cervids, the likelihood of human susceptibility seems low. Laboratory evidence has demonstrated a molecular barrier against such cross-species infection, based on the failure of abnormal cervid prions to efficiently convert normal human prions to the infectious state. Likewise, abnormal cervid prions don't easily convert normal cattle prions, suggesting that cattle won't get CWD and pass it on to humans who eat tainted beef. While cattle can contract CWD if inoculated with the infectious agent, long-term studies placing cattle in close proximity to diseased cervids have resulted in no cases of natural transmission. Williams summarizes what all this suggests: “Never say never, but based on the [molecular] work, the CDC's findings, and the epidemiology, we certainly don't have evidence that humans have gotten CWD.”

## 

**Figure 2:**
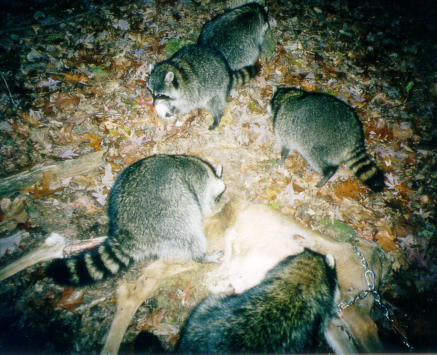
Identifying Animals at Risk from CWD A raccoon family feeds on a deer carcass staked out by researchers at the University of Wisconsin, in a study aimed at determining which species could be at risk of contracting CWD. (Photo courtesy of the Wisconsin Cooperative Wildlife Research Unit, University of Wisconsin-Madison.)

**Box 1. How Prions Confound Research**

The relative newness to science of CWD and mad cow disease is one reason they aren't well understood, but sheep scrapie was first identified in Great Britain in 1732—and it still isn't well-characterized. The main problem is the numerous roadblocks to researchers posed by prions, the disease agents of such TSEs.

Because normal and abnormal prions have identical amino acid sequences, the immune system neither recognizes an infection nor mounts a prion-specific response. Accordingly, an antibody specific to prions has not yet been identified. Without nucleic acid, prions can't be detected or analyzed using conventional techniques such as polymerase chain reaction. They also are extraordinarily resistant to a range of treatments that typically kill or inactivate infectious agents, such as ultraviolet and ionizing radiation, heating, and most chemical disinfectants. The infectious form is largely resistant to degradation by protease enzymes, and in laboratory animals it can incubate for months to years before clinical disease signs appear. Finally, prion diseases must compete for space in expensive, biohazard-safe labs. It's therefore unsurprising that knowledge of these diseases has not sped forward. Still, scientists hope that the recent upsurge of research into BSE, CWD, and scrapie in the United States and Europe will produce synergistic results for preventing and controlling all TSEs.

**Box 2. Who Else Might Get CWD?**

When mad cow disease broke out in the United Kingdom in the 1980s, cattle and humans were far from the only species found to be affected. Among other bovids in zoo and research colonies that contracted spongiform encephalopathy from tainted beef were nyala, gemsbok, eland, Arabian and scimitar-horned oryx, greater kudu, and North American bison. A feline version of the disease was found in domestic cats, cougars, cheetahs, ocelots, and tigers. Among primates, rhesus macaques and lemurs were also infected.

Unlike BSE, CWD is not thought to be transmitted through feed. But three species of cervids are naturally susceptible, and the question arises of how many other species might be in danger. To help answer that question, Michael Samuel and colleagues at the University of Wisconsin are staking out deer carcasses to see which scavengers come to feed. With flashlit photography, they've discovered “an amazing cast of characters,” including hawks, owls, crows, dogs, cats, coyotes, raccoons, skunks, mink, foxes, and opossums (Figure 2). Mammalian scavengers in the state's CWD-affected region will later be examined for disease.

## Abbreviations

BSE: bovine spongiform encephalopathy
CDC: Centers for Disease Control and Prevention
CJD: Creutzfeldt-Jacob disease
CWD: chronic wasting disease
TSE: transmissible spongiform encephalopathy
USDA: United States Department of Agriculture
